# Hemp Waste as a Substrate for *Hermetia illucens* (L.) (Diptera: Stratiomyidae) and *Tenebrio molitor* L. (Coleoptera: Tenebrionidae) Rearing

**DOI:** 10.3390/insects14020183

**Published:** 2023-02-13

**Authors:** Wael Yakti, Nadja Förster, Marcus Müller, Inga Mewis, Christian Ulrichs

**Affiliations:** Urban Plant Ecophysiology Division, Faculty of Life Sciences, Thaer-Institute of Agricultural and Horticultural Sciences, Humboldt-Universität zu Berlin, Lentzeallee 55, 14195 Berlin, Germany

**Keywords:** black soldier fly, yellow mealworm, cannabis, THC, agricultural wastes

## Abstract

**Simple Summary:**

Growing insects on agricultural wastes is an approach to reduce the amount of waste produced by farming operations and convert it into valuable products such as protein (for food and feed) and fertilisers. The larvae of two insects were fed with substrates that contain hemp residues (welted flowers, old leaves, and stalks) and were able to grow. The produced black soldier fly larvae did not contain the psychoactive ∆-9-tetrahydrocannabidiol (∆^9^-THC) and contained beneficial cannabinoids such as cannabidiol (CBD), and the amounts were minimal in comparison to the amount found in the initial substrate before given to the larvae. Mealworms were grown on hemp material that is rich in bioactive compounds, none of which was detected in the produced larvae. This study demonstrates that hemp agricultural wastes can be used as feed for these two insects, and the produced larvae do not contain the psychoactive compounds of hemp.

**Abstract:**

The proper treatment of cannabis agricultural wastes can reduce the environmental impact of its cultivation and generate valuable products. This study aimed to test the potential of cannabis agricultural wastes as a substrate for the rearing of black soldier fly larvae (BSFL) and yellow mealworms (MW). In the case of BSFL, replacing the fibre component (straw) in the substrate with the hemp waste can increase the nutritional value of the substrate and led to bigger larvae. The bigger larvae had lower P and Mg, and higher Fe and Ca. Crude protein also varied based on the size of larvae and/or the content of protein in the initial substrate, which was boosted by replacing straw with hemp material. No other cannabinoids than cannabidiolic acid (CBDA), cannabigerolic acid (CBGA), and cannabidiol (CBD) were found in significant amounts in the larvae. In the case of MW, the larvae grew less on the hemp material in comparison to wheat bran. Replacing wheat bran with the hemp material led to smaller larvae with higher Ca, Fe, K, and crude protein content, but lower Mg and P values. No cannabinoids were detected in the MW fed with the hemp material.

## 1. Introduction

*Cannabis sativa* is a flowering plant that originates from central Asia, and has been cultivated throughout history for its fibres, seeds, and oil [1,2]. Within the species *C. sativa* L., three varieties are traditionally known: *C. sativa* var. *sativa, C. sativa* var. *indica* and *C. sativa* var. *ruderalis*, which differ not only in their morphology but also in their phytochemistry [3]. The plant is well known to contain cannabinoids secondary metabolites such as the psychoactive ∆-9-tetrahydrocannabidiol (∆^9^-THC) and cannabidiol (CBD). Some certified cultivars in the European Union (EU) have a very low ∆^9^-THC content and contain mainly the nonintoxicating cannabidiol (CBD), which is now widely used in the treatment of various mental and psychiatric disorders [4]. The cultivation of the plant is expanding worldwide, and particularly in the EU, the cultivation areas almost doubled from year 2015 to year 2021 [5]. Aside from the use of seeds in nutrition [6], leaves and flowers can be a valuable source of bioactive cannabinoids while the remaining hemp by-products, i.e., the stalks, including fibres and shives, represent the majority of the plant′s dried weight and are low in cannabinoids [7]. Much of this fraction of plant biomass ends up as a low-value residue despite being a source of cellulose and other biomaterials [8]. Generally, processes that valorise agricultural residues are gaining an increasing attention due to economic and environmental consideration [9]. The efficient use of such residues can play a significant role in supporting bio-economy visions and shifting from linear to circular production systems [10].

Insects, such as the black soldier fly, *Hermetia illucens* (Diptera: Stratiomyidae), and the yellow mealworm, *Tenebrio molitor* (Coleoptera: Tenebrionidae), can be mass-reared on a wide range of substrates, including agricultural wastes [11,12,13,14,15]. The larvae of these insects or their downstream products can be used as animal feed [16,17] or as food in the case of the mealworm [18]. The regulations, however, differ among countries and allowing the use of insects as food or feed can change over time [19]. Both insects present a protein source that is more sustainable than fishmeal in aquaculture [20,21], provide an environmental enrichment for poultry [22], and could be used as a pig and cattle feed [23,24]. Additionally, the fat accumulating in the larval biomass can be extracted on an industrial scale and can be used in various applications such as biofuel production and in cosmetics [25,26], in addition to being a feed additive [27]. In comparison to conventional livestock, insects can be reared in a much smaller space, consume less water, and have a high growth rate [28,29], making them a more sustainable protein source.

It is known that the utilization of locally sourced agricultural by-products or waste as feed for insects can decrease the environmental impact of both agriculture and insect production, while also making insect production economically viable, even when accounting for the cost of insect rearing and when insect yield is compensated [11,29,30]. The hemp used in this study is grown to produce CBD-rich hemp tea, for which high quality fresh leaves and flowers are used, leaving a waste biomass of stalks and low-quality leaves (see materials and methods). This material is usually dumped in the field or composted. The potential of exploiting this side stream as a feeding substrate for the black soldier fly and mealworm larvae was assessed. In two separate feeding experiments with two insect species, larvae were grown on commonly used rearing substrates that were partially or fully substituted by the hemp material. The growth and partial nutrient composition, as well as the occurrence of hemp cannabinoid in the larval biomass were assessed. We hypothesised that the hemp material can be used as a substrate for insect rearing, and the insect’s biomass would accumulate bioactive cannabinoids.

## 2. Materials and Methods

### 2.1. Black Soldier Fly Colony

The black soldier fly (BSF) strain used in this study was obtained from the Leibniz-Institute of Freshwater Ecology and Inland Fisheries (IGB) (Berlin, Germany). The population receives chicken feed (K (11 4) o.K., Agravis Raiffeisen AG, Velten, Germany) and is reared according to Yakti et al. [31]. Before the experiment, eggs were collected manually from rearing cages and were let to hatch for 24 h on a substrate that contained 30% dry chicken feed and 70% water. The neonates were let to grow for 6 days and reached an average single-larva weight of 7 mg at the start of the experiment.

### 2.2. Yellow Mealworms Colony

Eggs of mealworm were gifted by Inagro Insect Research Centre (Rumbeke-Beitem, Roeselare, Belgium). 60 g of yellow mealworms eggs were placed on 600 g of wheat bran, and the mealworms were let to hatch and develop for 5 weeks. The mealworm larvae reached 5 mg each and were used in the experiment.

### 2.3. Hemp Material

The by-product of hemp tea production was delivered by Die Hanflinge company (Gumtow, Brandenburg, Germany). The material mainly consists of stalks/secondary stems of (*C. sativa* L. var. Futura) and low-quality (welting, yellow, damaged) leaves and buds. It did not contain the main stems or seeds. The plant materials were delivered air-dried, and were further dried in the oven (Heraeus Holding GmbH, Hanau, Germany) at 60 °C for 6 h and no weight loss was further observed. The materials were blended to a <2 mm particle size.

### 2.4. Black Soldier Fly Larvae Feeding Experiment

To investigate the potential use of the hemp material as a BSF rearing substrate, three substrate mixtures were prepared and are shown in Table 1.

The base feed mixture (H0) contained chicken feed (K (11 4) o.K., Agravis Raiffeisen AG, Velten, Germany) and a high proportion of straw (Table 1). The straw was substituted with the hemp materials in treatments H44 and H66. The substrates were prepared by adding water to the dry feed mixtures and then mixing with a kitchen spatula. Based on the composition of feed mixture (chicken feed, straw, and hemp) the contents of cannabinoids differed among treatments (Table 1). The mixtures were kept at room temperature for 6 h to let the dry components soak in water. For each treatment, 5 replicates of 500 g substrate were put in polyethylene boxes (area of 12 × 17 cm) each of which contained 700 larvae (7 days old with an average weight of 7 mg). The young larvae were quantified by counting and weighing 3 groups of 1000 larvae from the same batch, and the obtained average single-larva weight was used to calculate the weight of the groups. The larvae grew at 30 °C and in 30–40% rH. The growth was monitored on the 2nd and the 4th day by collecting and weighing 50–100 larvae. The larvae were put back in the box after determining the number and the weight of the collected larvae. Harvest took place on the 6th day as the single-larva weight dropped in treatment (H66). The harvested larvae, as well as the initial substrates, were stored at −80 °C, then lyophilised for the chemical analyses (see below).

### 2.5. Mealworm Feeding Experiment

The mealworm feeding experiment had four treatments and five replicates per treatments. Wheat bran (LM Lindenberger mill GmbH, Brandenburg, Germany) was substituted with the hemp in different proportions. The treatments were as follows (based on weight): T0 consisted of 100% wheat bran, T33 consisted of 67% wheat bran and 33% hemp, T66 consisted of 34% wheat bran and 66% hemp, and T100 consisted of 100% hemp. The mass fractions of cannabinoids in the hemp material used in this experiment is shown in Table 2. Each box contained 500 g substrate and 1750 larvae (5 weeks old, quantified as mentioned in the Black soldier fly larvae (BSFL) experiment). The larvae were grown for 5 weeks at 28 °C and 30–40% rH. Three pieces (1 cm^3^ each) of 20 g/L agar were added on the top of every box ad libitum so that the larvae always had access to a wet component. Dry and mouldy agar pieces were replaced. Starting from the 4th week, all the larvae were sieved from the T0 boxes and the rest substrate was quantified. The experiment was ended on the fifth week when less than 10% of the initial substrate was present. The larvae were processed as in the BSFL experiment for chemical analyses.

### 2.6. Determination of Growth and Biomass Parameters

In both experiments, the growth of larvae was monitored by weighing at least 15% of the total number of larvae per box, and the collected larvae were put back in the boxes after weighing. The total yield after and before lyophilisation was measured and total number of surviving larvae was obtained by dividing the total fresh yield by the single larvae weight obtained at the day of harvest.

### 2.7. Elemental Analysis and Protein Quantification

The lyophilised larvae and the initial substrate (before being fed to the larvae) were subjected to chemical analyses. The larval materials were blended manually using a mortar and pestle with the addition of liquid nitrogen. The substrates were ground by milling using Retsch MM 400 machine (Retsch GmbH, Haan, Germany). Nitrogen (N) (in the larval materials), phosphorus (P), calcium (Ca), magnesium (Mg), potassium (K), and Iron (Fe) were analysed as described in Yakti et al. [31]. The crude protein in the larvae was calculated using a protein-to-nitrogen conversion factor of 4.76 [32], and the Bradford protein assay was used to measure the protein content of the initial substrates. Table 3 shows the partial nutrient composition of the initial substrates.

For the extraction of total proteins in the substrate, 50 mg freeze dried and pulverised substrate material were used. As described in Jones et al. [33] and Mertens et al. [34], with some modifications, 1 mL 0.1 M phosphate buffer (pH 7.2; mixture of 800 mL 0.1 M NaH_2_PO_4_ and 150 mL Na_2_HPO_4_) was added to the sample. Samples were vortexed and incubated at room temperature for 30 min (vortexing was repeated from time to time). After centrifugation at 10,000 rpm for 5 min at room temperature, the supernatant was transferred into a new tube for further steps.

Protein concentration was measured using the Bradford Coomassie Blue assay method [35], where the colour change can directly be linked to the amount of protein in the extract. The measurements were done spectrophotometrically (UV mini—1240, Shimadzu, Japan) at 595 nm. A standard curve of serum albumin was used for quantification.

### 2.8. The Detection and Quantification of Cannabinoids

Cannabinoids were quantified in both the produced insects and the initial substrates. In the case of BSFL initial substrates, analysis samples were taken after the final wet mixed was prepared. In the case of the MW initial substrate, the main batch of dry hemp material used to prepare the mixes was homogenised and sampled. A modified method described by Mandrioli et al. [36] was used to extract and detect the cannabinoids. Briefly, 20 mg of lyophilised, pulverised substrate material (or, respectively, 100 mg for insect material) were extracted with 750 µL of extraction solution (methanol/chloroform 9/1, *v*/*v*) for 10 min at room temperature and 500 rpm on a shaker (Eppendorf SE, Hamburg, Germany). The samples were centrifuged (10,000 rpm, 5 min, room temperature) and the supernatant was collected in a glass vial. Thereafter, the pellet was re-extracted with 500 µL extraction solution twice. The combined supernatants were concentrated under nitrogen stream to dryness and refilled with 500 µL 100% acetonitrile. The extract was filtered using 0.22 µm SpinX tubes (Costar, Corning, New York, NY, USA), filled in HPLC vials, and stored at −20 °C until HPLC analysis.

The HPLC system consisted of a DIONEX P680, an ASI-100 auto sampler, a TCC-100 thermally-regulated column department, and an UltiMate 3000 Photodiode Array Detector. The software Chromeleon 7.2 was used for peak evaluation. All HPLC components including and the software were supplied by Thermo-Fisher (Thermo-Fisher Scientific, Dreieich, Germany). Reversed phase chromatography was carried out on an AcclaimTM RP18 column (3 μm, 120 Å, 2.1 × 250 mm, Thermo-Fisher). The eluents used for HPLC analysis were (A) 0.85% formic acid in ultrapure water and (B) 0.85% formic acid in 100% acetonitrile. The extracts were analysed with a flow rate of 0.4 µL/min at a column temperature of 35 °C, and the following gradient program: 70% B (0–3 min), 70–85% B (3–10 min), 85–95% B (10–17 min), 95–100% B (17–18 min), and 100- 70% B (18–28 min). The injection volume was 10 µL and peak detection was carried out at 265 nm. Commercially available standards of single compounds were used as references: cannabidiolic acid (CBDA), cannabigerolic acid (CBGA), cannabigerol (CBG), cannabidiol (CBD), ∆-9-tetrahydrocannabinol (∆^9^-THC), cannabichromene (CBC), ∆-9-tetrahydrocannabinolic acid (∆^9^-THC-A), and cannabichromenic acid (CBGA). The qualitative analysis and identification of cannabinoids was based on their retention times, specific UV-spectra and mass spectrometry (characteristic mass fragment ions by HPLC-DAD-ESI-MS3, [M-H]- and [M-H]+). The cannabinoid content was calculated in µg/g dry weight (DW), based on the calibration curve of the respective standard.

### 2.9. Statistical Analyses

The SPSS version 28.0.0.0 software (IBM Corp, New York, NY, USA) was used for the statistical analyses. Repeated-measure ANOVA (n = 5, *p* < 0.05) followed by Tukey’s HSD was used to compare the growth of the larvae over time between the different treatments. One-way ANOVA (n = 3–5, *p* < 0.05) was used to test the differences among treatments after approving data normality and the homogeneity of variance. Kruskal–Wallis test was used for parameters that did not meet the assumptions of the parametric test ANOVA. Sphericity of the data was also approved before conducting repeated-measures ANOVA, and Greenhouse–Geisser correction was used when the sphericity assumption was not met. Student′s *t* test was used to compare the mass fraction cannabinoids in the BSFL larvae.

## 3. Results

### 3.1. BSFL Feeding Experiment

**Figure 1:**
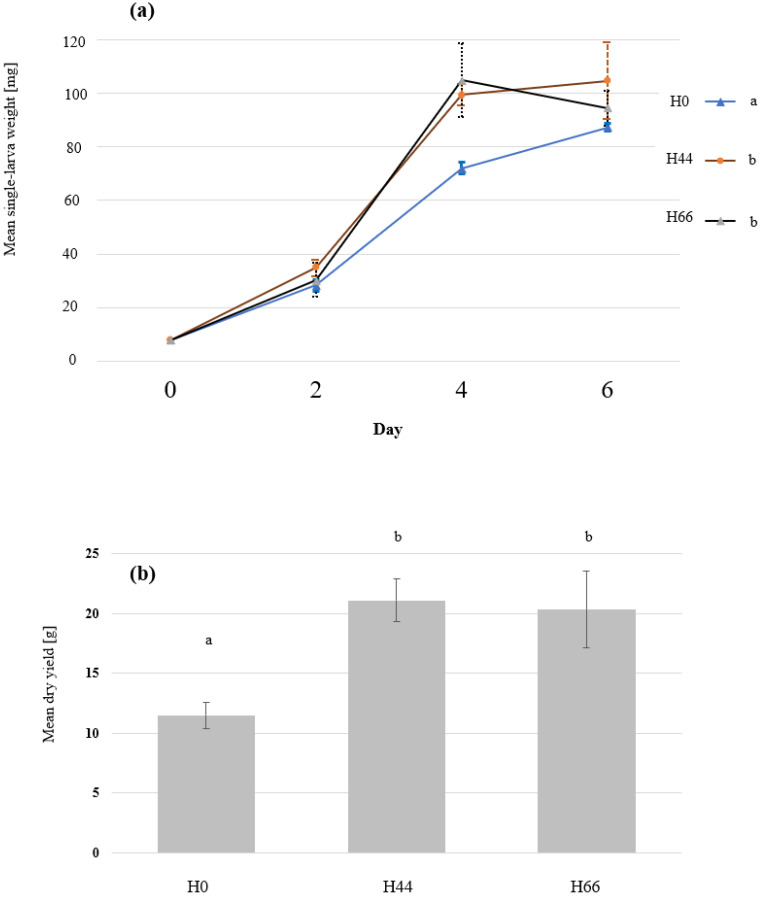
The growth and the final dry yield of black soldier fly larvae (BSFL) grown on substrates that contain different ratios of hemp material. BSFL were grown on substrates with 0% hemp (H0), 44% hemp (H44), 66% hemp (H66). The means and standard deviations of (**a**) the single-larva fresh weight over time and (**b**) the final dry yield are shown in the graph. Repeated measures ANOVA, and one-way ANOVA followed by Tukey’s test revealed significant differences in the larval growth over time (n = 5, *p* < 0.001, F = 26.8) and final dry yield (n = 5, *p* < 0.001, F = 28.5), respectively. Significant groups are indicated by the different letters.

**Figure 2:**
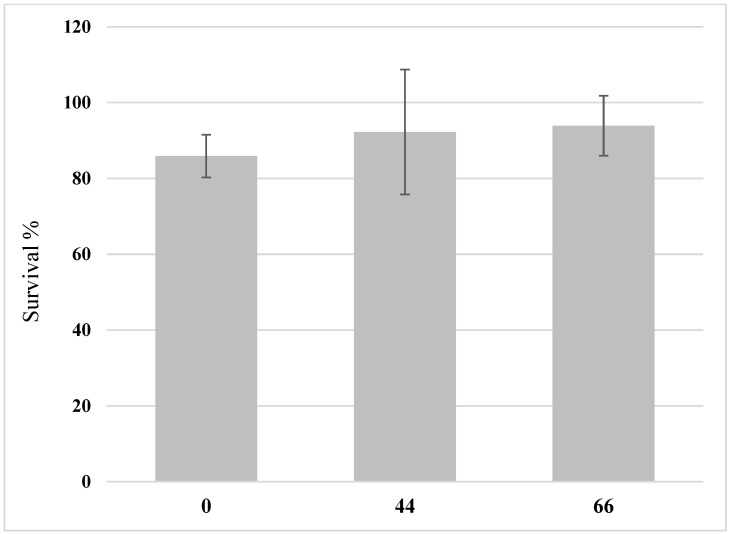
The survival of black soldier fly larvae (BSFL) grown on substrates that contain different ratios of hemp material. BSFL were grown on substrates with 0% hemp (H0), 44% hemp (H44), or 66% hemp (H66). The survival rate is shown in the graph with the standard deviations. One-way ANOVA (n = 5, *p* < 0.05) revealed no significant differences in survival between the treatments.

The larvae grew on all substrates and reached 78 to 104 mg/larva at the time of harvest (Figure 1). The highest growth was recorded in the treatments that contained both chicken feed and the hemp material (H44 and H66), while the larvae grew less in the chicken feed-straw substrate (H0) (Figure 1). The final fresh and dry yield obtained in the 6 days of growth also differed between the treatments as the yield in H44 and H66 exceeded that of H0 (Figure 1, only the dry yield is shown). The survival rate of the larvae ranged from 85% to 94% (Figure 2) and did not differ among treatments.

**Figure 3:**
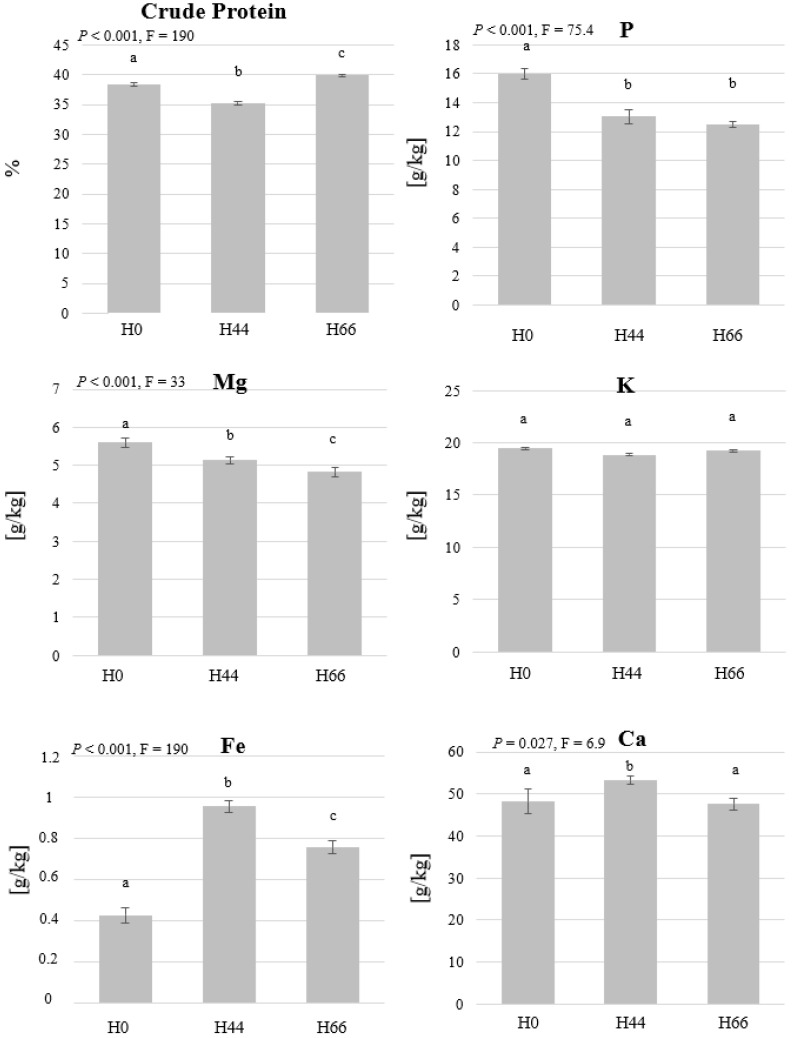
The mass fraction of protein, P, Mg, K, Fe, and Ca in dry black soldier fly larvae (BSFL) grown on substrates that contain different ratios of hemp material. BSFL were grown on substrates with 0% hemp (H0), 44% hemp (H44), or 66% hemp (H66). Shown are the means and the standard deviations of the measured mass fractions. One-way ANOVA (n = 3, *p* < 0.05) revealed significant differences between the treatments and the significant groups are represented by different letters.

The crude protein differed among the initial substrates and increased by the incorporation of hemp material into the substrate. The crude protein was the highest (39.8% of the dry biomass) in the larvae grown on chicken feed and hemp (H66, Figure 3). The crude protein values were lower in the larvae fed with the substrate that had straw, hemp, and chicken feed (H44). The mass fractions of all analysed elements in the larvae were also different among the treatments, with the exception of K (Figure 3). P, Ca Mg, and Fe accumulated in the BSFL while the mass fraction of K was lower in the larvae than in the initial substrates that contained hemp (H44 and H66). The mass fraction of P was the highest in the chicken feed-straw treatment (15.99 g/kg dry biomass) and decreased with the addition of hemp in the substrate. In the case of K, no differences were observed despite the different K content in the initial substrate. Significant differences were observed also in Ca and Mg as the values ranged between 53 and 48 g/kg, and 5.6 and 4.8 g/kg, respectively. Additionally, the mass fraction of Fe was the highest in H44 treatment (0.95 g/kg dry biomass) and the lowest in the treatment that had no hemp (0.42 g/kg dry biomass) (Figure 3).

**Figure 4:**
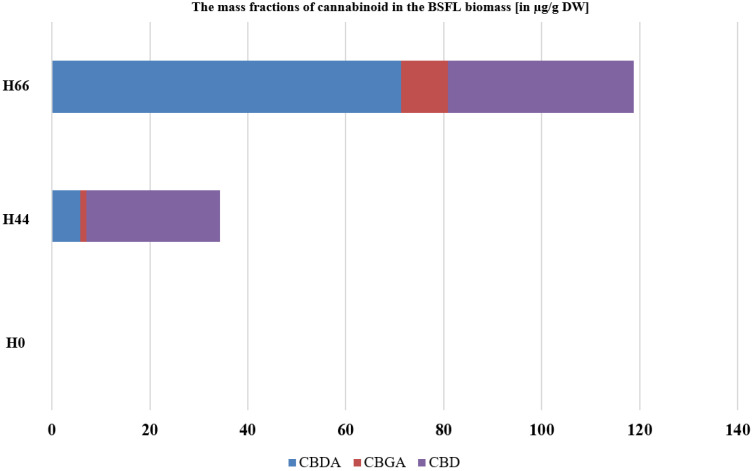
The mass fraction of cannabinoids in dry black soldier fly larvae (BSFL). BSFL were grown on substrates with 0% hemp (H0), 44% hemp (H44), or 66% hemp (H66). Shown is the mean cannabinoid mass fraction. The Student’s *t* test (n = 5, *p* < 0.05) revealed significant differences in the mass fraction of cannabidiolic acid (CBDA), cannabigerolic acid (CBGA), and cannabidiol (CBD). Only traces of CBC, CBCA, ∆^9^-THC, and THCA were detected in some samples.

Higher ratio of hemp material in the BSFL feeding substrate lead to higher cannabinoids mass fraction in the larvae. The highest mass fraction of cannabinoids was observed in the larvae grown on chicken feed and hemp substrate (H66) (Figure 4). Cannabidiolic acid, cannabigerolic acid (CBGA), and cannabidiol (CBD) were significantly more present in the H66 treatment. Only trace amounts of ∆-9-tetrahydrocannabinol (∆^9^-THC) and ∆-9-tetrahydrocannabinolic acid (∆^9^-THC-A) were detected and could not be quantified. Additionally, cannabichromene (CBC) and cannabichromenic acid (CBCA) were not detected in the larvae of all treatments and were therefore excluded.

### 3.2. Mealworm Feeding Experiment

**Figure 5:**
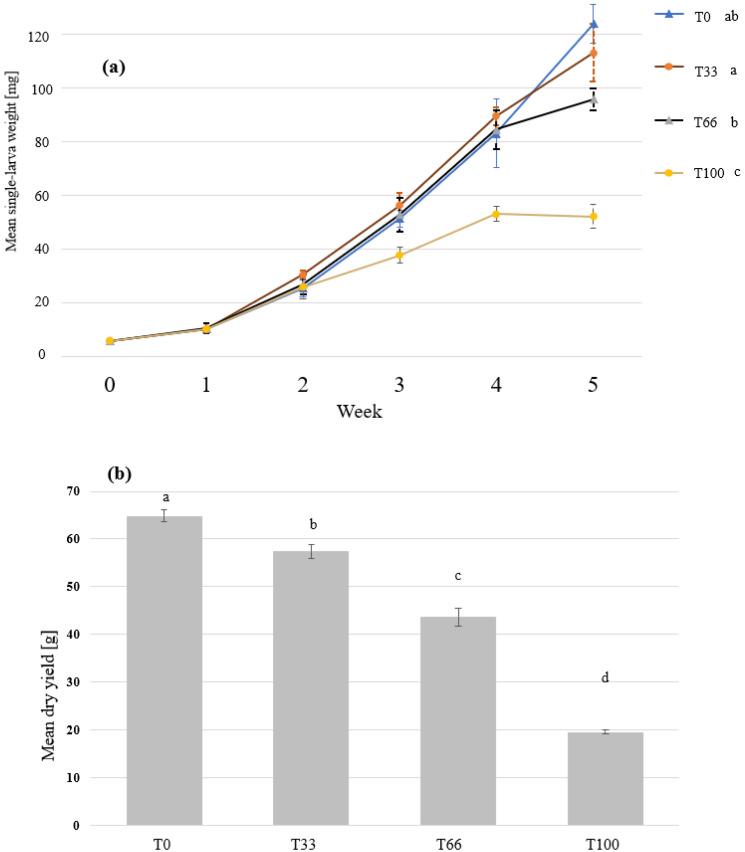
The growth and the final dry yield of mealworm larvae grown on substrates that contain different ratios of hemp material. Mealworm larvae were fed with wheat bran (T0), 67% bran and 33% hemp (T33), 34% bran and 66 hemp (T66), or 100% hemp (T100). The means and standard deviations of (**a**) the single-larva fresh weight over time and (**b**) the final dry yield are shown in the graph. Repeated measures ANOVA with Greenhouse–Geisser correction (n = 5, *p* < 0.001, F = 1531.7), and Kruskal-Wallis test (n = 5, *p* < 0.001) revealed significant differences in the larvae growth and final dry yield, respectively. Significant groups are indicated by the different letters.

**Figure 6:**
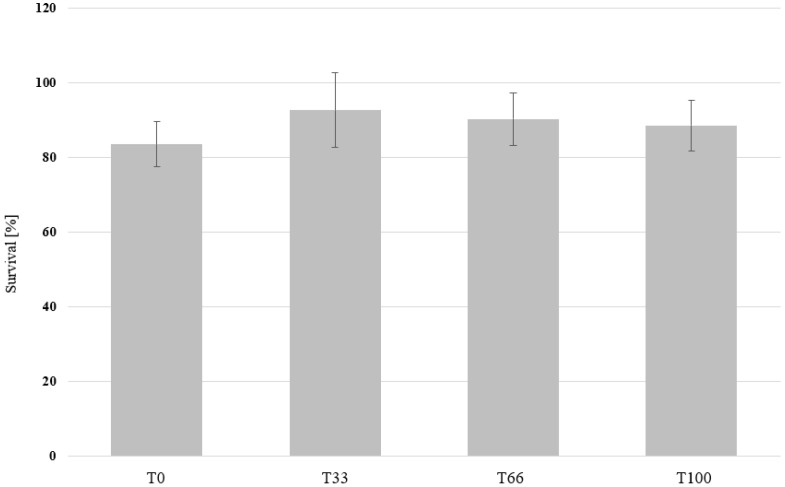
The survival of mealworm larvae grown on substrates that contain different ratios of hemp material. Mealworm larvae were fed with wheat bran (T0), 67% bran and 33% hemp (T33), 34% bran and 66 hemp (T66), or 100% hemp (T100). The survival rate is shown in the graph. One-way ANOVA (n = 5, *p* < 0.05) revealed no significant differences in survival among the treatments.

The mealworms (MW) grew for 5 weeks in all the boxes and the individual larval weight ranged from 53 to 123 mg for the different treatments (Figure 5). Significant differences in the growth rate and final dry yield were observed. The larvae performed the best when supplied with wheat bran (T0) treatment with a final dry yield of 64.8 g. The yield decreased with replacing wheat bran with the hemp material and was 19.61 g when fed hemp material only (T100). As in the BSFL feeding experiment, no differences in the larval survival were observed and the values ranged between 83.6 and 92.7% (Figure 6).

**Figure 7:**
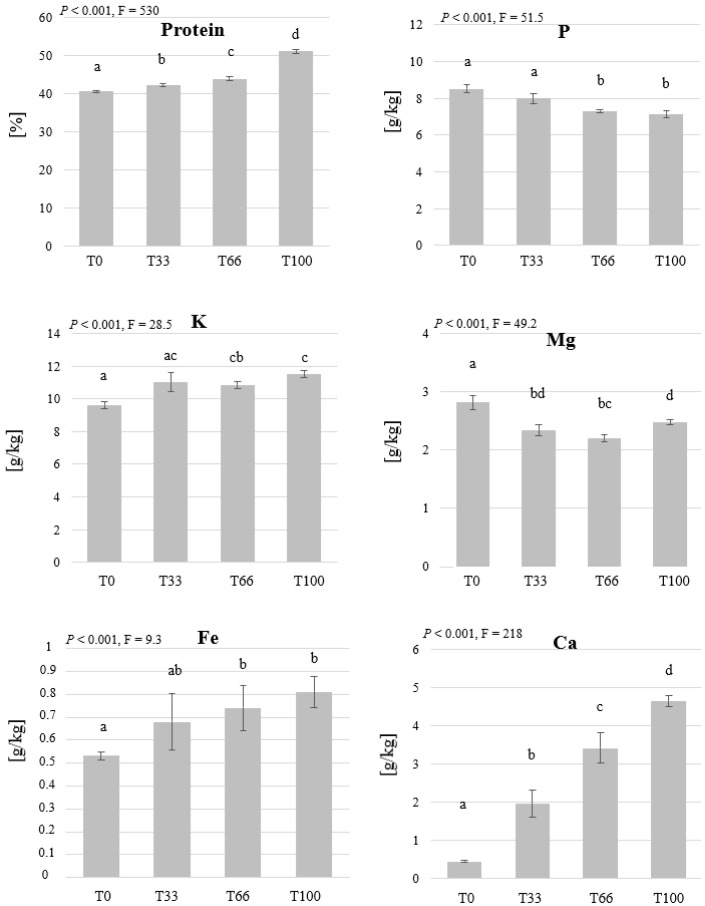
The mass fraction of protein, P, Mg, K, Fe, and Ca in mealworm larvae grown on substrates that contain different ratios of hemp material. Mealworm larvae were fed with wheat bran (T0), 67% bran and 33% hemp (T33), 34% bran and 66 hemp (T66), or 100% hemp (T100). The means and the standard deviations are shown in the graph. One-way ANOVA (n = 5, *p* < 0.05) revealed significant differences between the treatments in the crude protein and Fe mass fractions. Kruskal-Wallis test revealed significant differences in K, Mg, P, and Ca mass fractions. Significant groups are indicated by different letters.

In contrast to the BSFL, no accumulation of the analysed elements was observed in the MW larvae biomass except for P (Figure 7). The values of P were higher in the treatments that contained higher proportion of wheat bran (T0 and T33), which also had higher P mass fraction in the initial substrate (Table 3). Crude protein was the highest in the larvae fed on the hemp material (51.2%) and decreased with the decreasing the hemp content. A relation between the mass fractions of elements in the initial substrate and the larvae was observed in K, Ca, and Fe and the values differed significantly among the treatments. The mass fraction of Mg was the highest in the larvae fed with bran (T0) (2.81 g/kg dry biomass), which had the highest Mg values in the initial substrate.

The cannabinoids were also analysed in the mealworms but were not detected.

## 4. Discussion

The worldwide hemp market is projected to grow dramatically due to the industrial and pharmaceutical applications of the plant and its bioactive secondary metabolites [37]. As a crop produced with low environmental footprint [38], and low water and fertiliser use [39], its cultivation can have positive implications and can support bio-economy strategies [40]. However, while hemp has multiple uses, it is often cultivated for one or two specific purposes, which results in the generation of various residues, such as leaves, when cultivated for seeds or buds. Utilising this waste-stream directly as animal feed, despite its nutritional value [41], is prohibited in the European Union (EU) based on the current regulations [42]. This is mainly due to the presence of the psychoactive tetrahydrocannabinol (THC) in such materials. This study aimed, therefore, to test the growth of black soldier fly larvae (BSFL) and yellow mealworm fed fully or partially with a hemp agricultural wastes, and to determine if ∆^9^-THC and other cannabinoids accumulate or occur in the produced larval biomass.

In the presented study, BSFL grew on all the diets provided. The H0 treatment contained 12.8% chicken feed and a high content of straw (24.4%) to represent a low value organic substrate. The straw fraction was partially or fully replaced by the hemp material (in treatments H44 and H66). We hypothesised that replacing the nutrient-poor straw with the hemp material would improve BSFL growth and change their nutritional composition, which was confirmed in the results. BSFL are known to grow on nutrient-poor side streams and biowastes [43,44], but plant leaves, despite their high abundance, are not commonly used as rearing substrates given their low nutritional value and the occurrence of anti-nutrients as shown in the case of tomato leaves [45]. The dry mass of hemp leaves and flowers has been reported to contain 13 to 23% protein [41,46]. Additionally, the hemp fibre fraction consists mainly of cellulose (reviewed in Shahzad [47]) which provides a favourable physical properties to the substrate enhancing BSFL growth and survival [48]. This study shows that using hemp material to complement BSFL diets improves BSFL performance and yield (Figure 1).

Many factors can influence the survival of BSFL including substrate moisture [49], nutrient composition [50,51], rearing temperature [52,53], box size and larvae density [31,54], and the physical properties of substrates [48]. In the presented experiment, no differences in survival between the treatments were observed, and the values (over 80%) were higher than those observed in BSFL grown on agricultural side-streams such as winery by-product [55] or tomato leaves [45]. This indicates that the hemp material, despite being rich in bioactive compounds, did not contain significant amounts of insecticidal secondary metabolites as known for glycoalkaloids in *Solanacea* plants [56] or glucosinolates in the *Brassica* family [57].

In this work, the protein content in the BSFL was between 39.8% and 35.2%, which is in the range reported in other studies [58]. BSFL protein content has been shown to slightly differ based on the substrate provided to the larvae and to be more linked to the carbohydrate provided [59]. This, however, is not the case for larvae grown in lower densities on the same feed which leads to higher availability of proteins for a single larva [31,60]. The protein content of the initial substrate was enhanced when replacing the straw with the hemp material, but the protein content in the larvae, despite differing among the treatments, did not correlate with that of the substrate (Figure 3). The high protein content in treatment H0 can be explained by the low weight of the harvested larvae, as a trade-off between high growth and fat content, and BSFL protein content has already been reported in other studies [15,31,61]. This, however, does not explain the high protein content in the H66 treatment, which had the highest protein content. Larvae in treatment H66 lost weight between day 4 and day 6 (Figure 1), which could indicate that the larvae consumed some of their fat reserves leading to lower fat and higher percentage of protein in the harvested biomass. It could be that a maximum weight was achieved on day 5 before a starvation effect observed in day six. Nevertheless, the trade-off between substrate initial protein level, the fat content, and the starvation (or harvest time) of the larvae needs to be further investigated.

The mineral composition of BSFL highly depends on the feeding substrate [62]. The presented results show that P, Mg, Ca, and Fe accumulated in the harvested BSFL biomass (Figure 3), which has been also reported in other studies [31,63,64,65]. Crude protein and P mass-fraction have been shown to only differ marginally based on the provided BSFL diet [66]. In this work, however, BSFL P fraction did not differ based on the initial concentration of the substrate which contradicts with the observations of Liland et al. [67] who incorporated brown algae in different ratios into BSFL diets. The observed differences in the values of P this study seem to be due to a dilution effect as the value went lower for bigger larvae. The values of Mg and K observed in our study are higher than those observed by Chia et al. [65] and Spranghers et al. [68], probably due to the lower growth of larvae in the current study, as minerals such as Ca, Mg, and K could have lower concentration in bigger larvae (dilution effect) [31].

In the vast majority of animals, cannabinoids, both endogenous or exogenous, are able to activate cannabinoid binding (CB) receptors located mainly in the nervous system and the brain, and can regulate neurotransmission [69,70]. In insects, however, CB receptors are, most likely, the only absent mammalian neuroreceptor despite their presence in other invertebrates [71]. Nevertheless, the inclusion of cannabinoids in insects’ diet can lead to responses that differ among taxa. Park et al. [72] has reported positive and negative effects of CBD on *Manduca sexta*, whereas it helped the larvae against alcohol-induced stress and yielded in increased diet consumption (positive effects), but acted as a deterrent (negative effects). A trade-off has been observed in *Drosophila melanogaster* adults, as a preference for cannabinoids has been recorded in addition to an inhibition of food uptake [73]. In the current study, the initial substrates used contained a moderate level of cannabinoids (Table 1). The cannabinoids content in the produced BSFL biomass increased when the larvae were cultivated on substrates containing higher amounts. BSFL have been shown to accumulate health-relevant plant secondary metabolites such as α- and β-carotene [74] and to contain astaxanthin supplied in the substrate [75]. As shown in the results, the integration of hemp materials in this study leads to the production of insect biomass that had only trace amounts of ∆^9^-THC. In the EU, hemp-derived feedstuff should not contain more than 10 ppm of ∆^9^-THC, as such, only seeds-based materials are allowed as feed [42]. Cannabidiolic acid was present in the larvae, mainly in the treatment with high hemp material content (Figure 4). Cannabidiolic acid (CBDA) is considered an under-investigated compound despite its abundance in the cannabis biomass [76]. The presence of this compound in feedstuff could be beneficial for animals as it has been shown to reduce nausea and anxiety rats [77], in addition to possessing anti-inflammatory effects [78]. CBD was also detected in the produced BSFL and the difference, despite being significant, was lower than that of other cannabinoids (37.9 µg/g in the H66 treatment vs. 27.24 µg/g in the H44 treatment). CBD is considered as a compound with high potential in veterinary medical applications and its presence can confer benefits for animal health [79]. Nevertheless, more research is needed to get more insights into the occurrence of cannabinoids in the BSFL fed with hemp agricultural wastes.

Mealworm larvae grew the best in wheat bran and the growth was significantly reduced with the incorporation of hemp material (Figure 5). Wheat bran is considered as a common substrate for rearing MW [80,81], and the performance has been shown to decrease when the substrate is substituted by agricultural side-streams [30]. This, however, could still provide an economical benefit when the agricultural side stream is of a much lower value and higher availability. It is important to note that the hemp material provided a nutritional value for the mealworms as growth was only decreased by 12% when replacing 34% of the wheat bran with hemp (T0 and T66, Figure 5). The lower growth in the T100 treatment can be attributed to the lower content of macronutrients, such as proteins and carbohydrates, in the hemp material in comparison to the wheat bran (T0).

In the MW experiment, the mortality did not differ among the treatments and survival ranged between 83.6 and 92.7% (Figure 6). These values are comparable to studies that used wheat bran as a base feed for MW, and in the same range of temperatures used in our study [11,82,83]. However, lower survival values can be expected in unbalanced diets as shown by Oonincx et al. [66] on high-fat, low-protein diets.

The protein content in mealworm larvae was the lowest in the T0 and the highest in the T100 treatment. Differences in crude protein values were also observed by Harsányi et al. [84] based on the feed given, who also observed crude protein values in the range of our study. The range of crude protein values observed in this study are in accordance to published studies such as the work of González et al. [85] and Ravzanaadii et al. [86] who reported larval protein contents of 48.8% and 46.4% in substrates that contain wheat bran and vegetables, respectively. In our study, the reduction of crude protein in the larvae that was observed in the BSFL as well as in the mealworm trial, can be attributed to the dilution effect, as bigger larvae can accumulate more fats, leading to the reduction of protein percentage in the insect biomass.

The mineral composition of mealworms has been shown to be dictated by the composition of substrates used [87]. The concentrations of Ca, Fe, K, and P have been reported to differ between different stages of MW larvae besides a correlation between the concentration of elements in the feeding substrate and the larval biomass [88], this is in accordance to this study as such relationship can be clearly seen in case of Ca, Fe, K, Mg, and P (Figure 7 and Table 3).

In comparison to wheat bran, the hemp material contained higher concentration of Fe, Ca, K, but lower P and Mg, and proteins. Generally, Ca is known to occur in a low quantity in insects, the black soldier fly, however, is known to accumulate Ca in its exoskeleton [68,89] and therefore the accumulation was observed in BSFL but not in MW.

In contrary to BSFL, no cannabinoids were detected in the mealworm larvae despite the content of cannabinoids in the initial substrate. It is known that digestion and absorption pattern differ greatly between insect taxa [90], in addition to functional and morphological differences in coleopteran and dipteran mouthparts [91]. Water in semiliquid substrate that is used to rear BSFL could also be a reason for the occurrence of cannabinoids in the larval biomass. Despite being poorly soluble in water [92], this solubility could enhance the uptake of cannabinoids. Nevertheless, the factors influencing the uptake and accumulation of cannabinoids and other plant secondary metabolites in the larvae are a topic for future research.

This study suggests that using hemp agricultural waste as a feed source for BSFL and mealworms can produce a larval biomass with reduced cannabinoid content. However, regulations in the EU treat insect rearing in a similar manner to traditional livestock, including guidelines for acceptable substrates or feeds. These restrictions are expected to be eased for insects [93], and they differ between country and the animal they are intended to be fed to [19].

## Figures and Tables

**Table 1:** The feed composition and the cannabinoids mass fraction [µg/g DW] in the different treatments of the black soldier fly larvae experiment.

T	Substrate Composition (Weight-Based)				The Mass Fraction of Cannabinoids [in µg/g DW]								
	CF [%]	Water [%]	Straw [%]	Hemp [%]	CBDA	CBGA	CBG	CBD	∆^9^-THC	CBC	∆^9^-THC-A	CBGA	TC
H0	12.85	63.73	24.42	0	0	0	0	0	0	0	0	0	0
H44	12.85	63.73	8.14	16.3	219.7	36.5	8.3	100.5	2.29	23.7	8.9	36.9	436.0
H66	12.85	63.73	0	24.4	310.4	46.1	12.2	132	3.53	31.4	12.4	49.7	597.6

T: Treatment, CF: Chicken feed, TC: Total cannabinoid content in the dry mass, CBDA: cannabidiolic acid, CBGA: cannabigerolic acid, CBG: cannabigerol, CBD: cannabidiol, ∆^9^-THC: ∆-9-tetrahydrocannabinol, CBC: cannabichromene, ∆^9^-THC-A: ∆-9-tetrahydrocannabinolic acid, CBGA: cannabichromenic acid.

**Table 2:** Cannabinoids mass fraction [µg/g DW] in the hemp material used for the mealworm experiment.

Cannabidiolic acid (CBDA)	1030.68
Cannabigerolic acid (CBGA)	134.21
Cannabigerol (CBG)	18.85
Cannabidiol (CBD)	642.98
∆-9-tetrahydrocannabinol (∆^9^-THC)	14.94
Cannabichromene (CBC)	67.18
∆-9-tetrahydrocannabinolic acid (∆^9^-THC-A)	46.54
Cannabichromenic acid (CBCA)	109.62
Total cannabinoids	2064.99

**Table 3:** The nutrient composition of the initial substrates used in both feeding experiments.

Feeding Experiment	Treatment	Ca	Fe	K	Mg	P	Protein
		g/kg	g/kg	g/kg	g/kg	g/kg	%
BSFL experiment	H0	14.89	0.27	9.51	1.37	3.93	5.41
	H44	20.10	0.65	20.98	1.60	5.18	7.58
	H66	22.43	0.78	26.55	1.88	5.65	9.02
MW experiment	T0	1.07	0.16	15.38	5.44	12.71	10.82
	T33	9.19	0.19	19.13	5.25	10.07	9.82
	T66	17.62	0.21	22.00	5.12	7.03	8.89
	T100	26.06	0.23	25.79	4.97	4.10	7.9

## Data Availability

The data presented in this study are available on request from the corresponding author.
